# Aesthetic Rehabilitation Following Ocular Trauma in a Child

**Published:** 2013-11-18

**Authors:** Himanshi Aggarwal, Pradeep Kumar, Raghuwar D Singh, Varun Baslas

**Affiliations:** Department of Prosthodontics, King George’s Medical University UP, Lucknow, India

**Dear Sir,**

Pediatric ocular trauma is a commonly seen in patients with maxillofacial trauma.[1] It often results in phthisis bulbi, a small, shrunken, non-functional eye. Therapeutic options are limited in phthisical eyes and provide only symptomatic relief. Majority of patients with phthisis bulbi eventually become blind resulting in aesthetic disfigurement of face resulting in psychological and emotional trauma as well.[2]

A 2½-year-old child presented with shrunken and scarred right eye with white patch (Fig. 1). Six months ago the child had a trauma with pencil. Clinical examination revealed enophthalmic and phthisical globe with corneal opacity and normal sclera without any residual vision. The treatment plan was formulated and explained to the parents to gain their cooperation. Before making the impression, custom impression tray was made by using autopolymerising polymethylmethacrylate (PMMA; Trevalon, Dentsply India Pvt. Ltd., Gurgaon, India) in dough stage as described by Allen and Webster.[3] The perforated tray was checked in patient’s eye socket and adjusted accordingly. A syringe without the needle portion was attached to the centre of impression tray to serve as a handle for the tray which also served to inject the impression material into the defect area to record the intra-orbital topography (Fig. 2A). The impression of the right eye was made with thin mix of ophthalmic grade alginate (Opthalmicmoldite, Milton Roy Co. Sarasota Fla.) and a two-piece dental stone (Kalabhai Karson Pvt. Ltd., Mumbai, India) cast was poured (Fig. 2B, C). The wax pattern with iris obtained from exactly matching stock eye was formed and tried in the patient’s eye. After making necessary modifications, the wax pattern was invested; flasked and de-waxing was done (Fig. 3A). The clear heat cured PMMA was packed in the dewaxed mould in dough stage, followed by routine finishing and polishing (Fig. 3B, 3C). The polished prosthesis was inserted in the socket after disinfection and lubrication with an ophthalmic lubricant (Fig. 4). Instructions were given to the patient regarding proper handling, insertion, removal and maintenance of the prosthesis. Patient was recalled after 24hours, 1week and 3months, and later 6 monthly.

**Figure:**
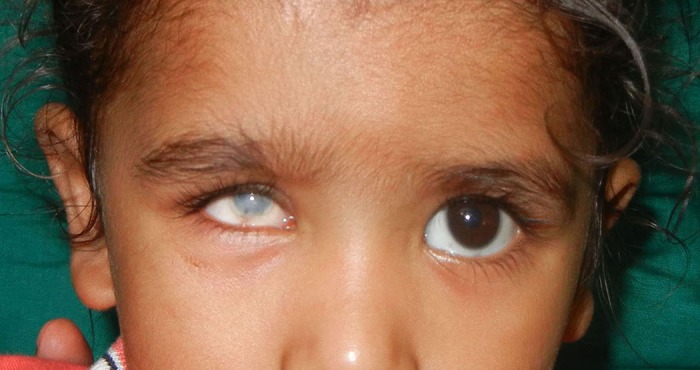
Figure 1: Phthisis bulbi of right eye with normal scleral colour.

**Figure:**
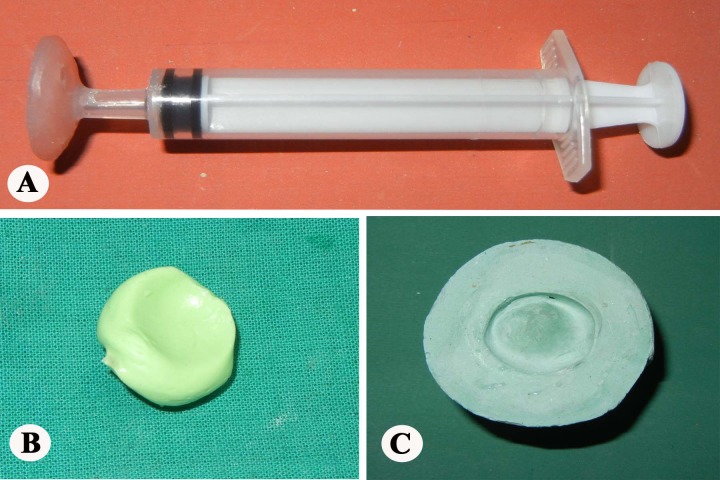
Figure 2: Syringe with attached perforated custom tray (A), impression of the ocular defect (B), and cast obtained after double-pour technique (C).

**Figure:**
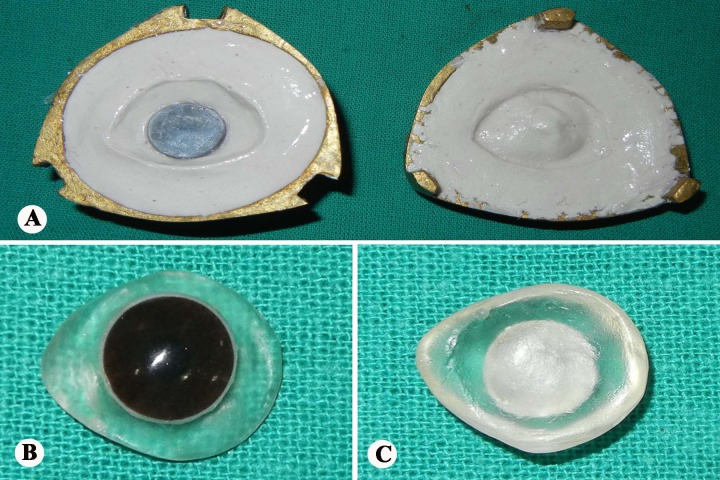
Figure 3: Dewaxed mould (A), transparent scleral shell with iris outer (B), and intaglio surface (C).

**Figure:**
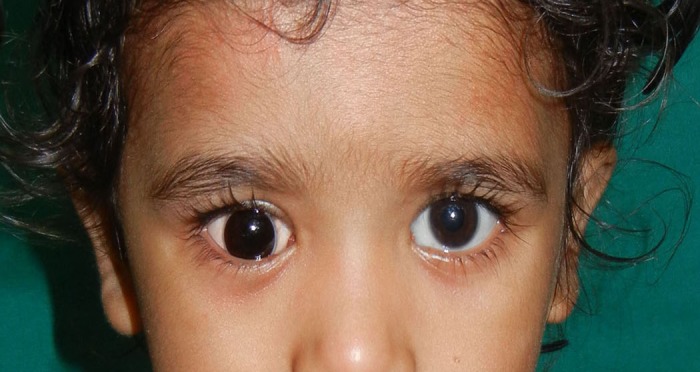
Figure 4: Transparent scleral shell fitted over phthisical right eye.

The phthisis bulbi can range from just corneal opacity without disfigured sclera to severe atrophy of ocular structures with disfigured sclera and loss of orbit fat. Once it is established that there is only enophthalmos with no disfigurement of sclera in either colour or contour, then, clear/transparent scleral shell prosthesis can be fitted over the phthisical globe. Prosthetic rehabilitation over the residual eyeball is the preferred treatment of choice over surgical intervention such as enucleation or evisceration.[4] The distinct advantages of clear scleral shell prosthesis over conventional scleral shade matched prosthesis include elimination of the time consuming, painstaking and technique sensitive steps of matching scleral shade; alleviation of the need of adding any veins for simulation of conjunctival vessel markings as natural conjunctival vessels can be seen through the transparent scleral shell; and at the same time, it is possible to continuously monitor the health of the underlying ocular structures through the transparent scleral shell. In the present case, the sclera was normal in color and contour, so it seemed logical to utilize the natural scleral shade by fabricating transparent acrylic shell prosthesis as contrary to the conventional method of fabrication where acrylic matching the patient’s sclera of normal eye is used.[5] At 3-months follow up, no complication with regard to health of underlying residual ocular tissues was found. Parents reported that the highly cosmetic ocular prosthesis has helped their child to successfully resume the socialization.

## Footnotes

**Source of Support:** Nil

**Conflict of Interest:** None declared

